# Minimizing Radiation in the Modern Electrophysiology Laboratory

**DOI:** 10.19102/icrm.2018.090805

**Published:** 2018-08-15

**Authors:** Albert J. Rogers, Chad R. Brodt

**Affiliations:** ^1^Department of Cardiovascular Medicine, Stanford University, Stanford, CA, USA

**Keywords:** Ablation, fluoroscopy, laboratory, radiation, safety

## Abstract

Historically, the electrophysiology laboratory has relied heavily on the use of ionizing radiation in the form of fluoroscopy for a broad range of interventions and diagnostics. As the harmful effects of radiation have become increasingly recognized and procedural technologies have advanced, electrophysiologists have adopted new workflows. The purpose of this article is to review the available literature and experience in minimizing radiation in the modern electrophysiology laboratory. This review first covers general approaches to reducing fluoroscopy radiation in the electrophysiology suite, with concepts that apply across all procedure types. These include the reduction of infrared emission through fastidious fluoroscopy settings, new and proven solutions for radiation shielding, and methods of creating distance between the radiation source and the operator to reduce exposure. Following this discussion, we review specific task-based techniques for reducing radiation during special electrophysiologic procedures and workflows such as vascular access, coronary sinus lead placement, catheter manipulation, and periprocedural planning studies.

## Introduction

For the general population, medical and health-related ionizing radiation dwarfs the degree of exposure attributed to natural radiation. For physicians, technicians, and other staff working in a cardiovascular interventional laboratory, however, the levels of radiation present far exceed those experienced by the general population. It has been estimated that exposure in the cardiac catheterization laboratory is roughly 5 mSv, or the equivalent of 250 chest X-rays, annually.^1^ The National Council on Radiation Protection and Measurements has based maximum permissible doses of radiation for those working in a cardiovascular interventional laboratory on those of other “safe professions”; however, the risk of malignancy, cataracts, dermatitis, thyroid dysfunction, and birth defects attributable to these levels of radiation are poorly quantified. Because doses less than those permitted by regulation are not devoid of risk, the radiation workforce has adopted a policy of limiting ionizing radiation to “as low as reasonably achievable” (ALARA).^2^ As interventional procedures continue to increase in complexity and the technologies to limit radiation usage and exposure evolve, the reality of ALARA is constantly in flux. The range of operator comfort with new technologies and the variable access of a laboratory to the newest available equipment creates a wide range for ALARA potential. This narrative review aims to discuss the potential hazards of ionizing radiation exposure, general approaches for decreasing fluoroscopy, and the experience with specific task-based alternatives to fluoroscopy. For the electrophysiologist with a diversified practice portfolio, the techniques described herein are available to reduce the exposure of ionizing radiation to patients, providers, and staff in the modern electrophysiology (EP) laboratory.

It has been well-known since the days of Marie Curie that the use of X-ray technology is accompanied by hazards. High energy photons (X-rays) enter the cell nuclei and deliver energy to form free radicals, which directly damage the deoxyribonucleic acid backbone. Repair mechanisms for double-strand breakages are imperfect and may introduce deleterious mutations or trigger cell death. According to 2008 recommendations from the International Commission on Radiological Protection (ICRP), the damage induced by radiation is estimated to cause a 5.5% increase in fatal malignancy per Sv when averaged across the population. The risk coefficient for cancer induction in patients is almost double compared with that in medical staff due to the concentrated exposure of radiation in time, with damage being out of pace with cellular repair mechanisms (10% versus 5.5% per Sv of effective dose).^3^ Radiation doses and times are calculated in a variety of ways across the literature but, when normalized to the rate above and unitized for time, fatal malignancy rates per hour of fluoroscopy range from 294 to 2,642 per million simple or complex catheter ablations.^4^ Small case studies of glioblastoma have increased concerns among cardiac interventionalists, although these have not conclusively shown causation.^5,6^ Low-fluoroscopy techniques have been emphasized for use in women of child-bearing age and in children because of their increased vulnerability to the effects of radiation. Obese patients also represent a vulnerable population due to the increased energy levels required to penetrate the soft tissue and the existence of greater body surface area for absorption. One study suggested that obesity potentially increases exposure up to double the normal amount.^7^

Cataract formation from radiation exposure similarly displays a range of reported values for dose thresholds for new cataracts. It is now recommended by the ICRP that 20 mSv per year exposure for the lens of the eye, with no single year exceeding 50 mSv, is adequate as a safety maximum. For reference, 20 mSv is approximately 155 standard EP procedures requiring 40 minutes of fluoroscopy, which is often exceeded in common clinical practice.^8,9^

Outside of malignancy, other risks include dermatitis, thyroid dysfunction, and germline mutations causing congenital abnormalities in the offspring of operators and staff.

## General approaches for reducing fluoroscopy

### Reduction of infrared emission

It is clear that the simplest way to reduce exposure to ionizing radiation from fluoroscopy is to use the pedal less often. However, even in circumstances in which fluoroscopy must be used, radiation emission can be minimized by decreasing the energy per pulse and the number of pulses per second and by collimating the beam. To generate real-time fluoroscopy video, pulses of X-rays are emitted, collimated, and collected several times per second. Catheters and cardiac structures absorb or scatter the X-rays and their shadows create images on the receptor and image intensifier. A grid above the receptor removes scatter (X-rays not parallel to the column). Each of these pictures is displayed in real time to show the relative positions of catheters and cardiac structures. Adequate X-ray energy is required for the penetration of airspace and the patient’s chest to generate contrast on the image and an adequate number of pictures per second is required to track moving objects over time.

Catheter tracking during procedures requires precise movements, but rapid movements are rarely used. Therefore, standard or factory-set frame rates may be beyond what is required for procedural efficiency and accuracy. Standard or default frame rates may range from 12 frames to 20 frames per second (fps). Some centers have decreased settings to as low as 2 fps without evidence of an increase in complications or prolongation of the procedure.^10^ An order of magnitude reduction in radiation dose may be achieved with an aggressive reduction in frame rate.

The fluoroscopy grid is a secondary radiation grid that is placed in front of the detector and which serves to remove scatter and sharpen the image. The removal of the fluoroscopy grid reduces the required exposure needed in order to create the image. This technique was studied using a radiation phantom model, resulting in an approximate 50% reduction in radiation dose.^11^ In the case of decreased visibility, fluoroscopy grids can be replaced within seconds if improved contrast is required for image quality.

Removal of the secondary radiation grid along with programming of a low pulsed fluoroscopy rate was associated with a two-thirds reduction in the excess fatal malignancy rate in patients undergoing simple and complex ablations. Fluoroscopy grids had to be related in a very small minority of cases.^4^

A recent survey performed by the European Heart Rhythm Association (EHRA) Electrophysiology Research Network in 2015 showed that only 50% of participating centers employed low-frame-rate techniques for reducing radiation.^12^ These measures represent low-hanging fruit, as they do not require equipment upgrades, special training, or significant changes to the procedural workflow; however, adoption rates of these techniques remain low.

### Shielding

Common shielding techniques in fluoroscopic procedures that have wide adoption include lead gowns, glasses, and thyroid collars as well as pull-down shields. These measures decrease the level of radiation exposure to the operator and laboratory staff but do not reduce the level of exposure to the patient. It is important to perform routine maintenance of lead equipment so as to assure safety. The gowns and pull-down shields also generate new problems. Pull-down shields can be problematic in device implantation procedures due to the positioning of the workspace and the radiation source. There is a striking incidence of orthopedic problems reported by interventional cardiologists that increases significantly in the years postfellowship and by up to 60% after a 20-year career.^13^ In the EP laboratory, procedures may be complex and nuanced, requiring prolonged procedure duration. Operator fatigue must be considered at the extremes, where extra weight and the restriction of movement imparted by lead shielding may impact the energy of the operator in a late case.

A suspended radiation protection system has been evaluated to enhance operator protection over the standard lead apron with respect to radiation exposure while also reducing orthopedic impact (ZeroGravity; Biotronik Inc., Berlin, Germany). In standardized dosimeter testing in a mock setup, the apparatus was shown to greatly reduce radiation across the body from 16- (gonads) to 78 (left axilla)-fold.^14^ Much more modest results were obtained from a prospective series of neurointerventional procedures involving a biplane fluoroscope. Although the impact on procedure time and complications has not been rigorously studied, the device is reportedly weightless and does not limit lateral movement or rotation, but does inhibit visualization of the pedals due to the limitation of trunk flexion. Maintaining the sterility of the device adds several additional minutes to the preparatory time between cases.^15^

Lead-free disposable drapes have been engineered to shield against scattered radiation without impinging on the operating field of view or compromising sterility. Placing the drapes between the operator and the patient reduces scatter radiation by 50% to 80% for the operator^16,17^; however, it may not provide similar safety for other staff and does not reduce exposure to the patient. Additionally, an 80% reduction in radiation is not adequate to remove lead vests and aprons, so this measure would not substantially impact the orthopedic consequences of working in the interventional laboratory.

Lead gloves, lead glass cabins or suspension systems, and radiation-absorbing (RAD) pads are still infrequently adopted, although these items remain some of the lower-cost options. RAD pads exist to reduce scatter from the patient to operator and should be placed on the patient, preferably draped from the top of their body to the table without obstructing the primary beam. Possible reasons for underutilization include the added burden of the materials and maintaining sterility, the localized benefit for the operator but not the staff or patient, and the patchy or imperfect nature of coverage.

### Distance

The dispersion of ionizing radiation decreases rapidly as a function of distance; therefore, creating distance between the fluoroscope and the operator and staff will reduce exposure for radiation workers. When procedures may be safely conducted in various projections, choosing those with the least exposure for the operator can significantly reduce total exposure. The left anterior oblique projection is most commonly used for ablation lesions according to the recent EHRA survey; however, this is the plane in which the operator is exposed to the greatest level of radiation.

Several companies have developed robotic catheter controllers to remove the risk of operator exposure by relocating them to the control room or beyond. By reducing their radiation exposure to zero, the provider also avoids wearing lead gowns and, by controlling the catheter with a mouse or joystick, they may opt to sit at a desk rather than to stand at the table.

The Stereotaxis Epoch^®^ solution (Stereotaxis, St. Louis, MO, USA) utilizes two electromagnets and a robotic catheter and sheath system. The system is coordinated with the anatomical mapping software for real-time localization of the catheter. To achieve precise movements of the catheter tip, the robotic sheath and catheter system can extend or retract the devices while deployed in the vasculature. For additional degrees of freedom, the electromagnets impart a magnetic movement to the catheter tip to deflect it into position. Using feedback from the mapping system, the robotic arm may move to predefined locations, thus executing a planned pattern. Safety studies have suggested similar safety profiles as those exhibited by manual catheter manipulation with successful completion of the procedure.^18,19^ The procedure has effectively reduced fluoroscopy exposure to the patient operator by pairing the navigation and mapping systems. Commonly reported patient and operator exposure times have been said to be as low as 12.81 minutes and 5.16 minutes, respectively.^20^ Initially, prolonged setup and procedure times limited the device’s use, but newer versions have been shown to significantly reduce procedure times.^21^

Hansen Medical (Mountain View, CA, USA) offers a deflectable catheter (the Sensei catheter system) that allows for millimeter precision in manipulations controlled by the operator through a computer interface. Movements are tracked using a three-dimensional (3D) anatomical mapping system. To date, however, experience with the Sensei robotic catheter system (Hansen Medical, Mountain View, CA, USA) has not been extensively discussed in comparison with the Stereotaxis Epoch^®^ solution (Stereotaxis, St. Louis, MO, USA).

Robotic catheter navigation systems reduce or eliminate radiation exposure to operators, but, often, staff must remain in the operating space in order to support the patient and maintain the robotic system. Similarly, this method does not directly decrease radiation exposure for the patient. The significant infrastructure requirements, learning curve, and training that are required in order to establish a robotic EP laboratory may explain why the adoption of these systems has tapered in the last two to three years.^12^

## Specific task-based alternatives

The potential for the highest dosages of radiation occurs during biventricular device implantation, pulmonary vein isolation (PVI), complex atrial ablation, and ventricular arrhythmia ablation. There is far less radiation incurred for pacemaker implantation or revision, EP study, or standard radiofrequency ablation, although variances are wide between reported doses.^22^

### Vascular access

Access to the subclavian, cephalic, or axillary artery is achieved in several ways. Cephalic vein isolation requires access via dissection and the vessel may not be of sufficient size for all the required leads, which can result in limitations due to multiple leads interacting. Blind subclavian access risks chest cavity or arterial puncture, while venogram-guided venipuncture requires exposure to contrast dye. Ultrasound has been applied in axillary venous access for devices and was found to be quick to learn, to reduce fluoroscopy exposure, and to achieve faster time to lead placement.^23^ It also seems that recent trainees have been taught general vascular access via ultrasound guidance prior to cardiovascular-specific training and may be able to adopt this technique more easily when compared with those who have been taught more traditional access approaches.

### Coronary sinus lead placement

Biventricular pacing has contributed significantly to multimodality heart failure therapy. Its application is successful in most cases; however, placing coronary sinus leads continues to challenge even the most experienced operators. Procedure times remain highly variable and significantly increased as compared with those of dual-chamber devices due to the highly variable coronary sinus and coronary venous anatomy. Fluoroscopy is used for the manipulation of the coronary sinus sheath, venograms, the manipulation of guidewires, and advancement and deployment of the left ventricular lead. Significant exposure to ionizing radiation occurs during this procedure due to the position of the operator next to the collimator and the difficulty of shielding. Very few technologies have specifically attempted to reduce radiation exposure during biventricular lead placement.

In a single-center cohort study, MediGuide™ (Abbott Laboratories, Chicago, IL, USA) and a low-fluoroscopy settings protocol resulted in a 96% reduction in ionizing radiation when compared with standard settings without MediGuide™ (Abbott Laboratories, Chicago, IL, USA) tracking. Individually, an 82% reduction in infrared exposure was achieved with MediGuide™ (Abbott Laboratories, Chicago, IL, USA) alone and a 60% reduction was achieved with low-fluoroscopy settings alone. Overall, procedure time with sensor-based navigation was decreased by 22%.^24^

Biventricular pacing remains one of the procedures that utilizes fluoroscopy most in the modern EP laboratory. The current methods and technology for the placement of biventricular leads leave a significant opportunity for further innovation.

### Transseptal puncture

Because of the difficulty of the procedure and the gravity of adverse outcomes that may occur following inaccurate performance, the transseptal puncture remains reliant on fluoroscopy in many cases. However, intracardiac echocardiography (ICE)-guided procedures have been developed to successfully achieve transseptal puncture without an increase in adverse outcomes. Ferguson et al. described a series of views, including the left innominate vein with rotational ICE, that allow for safe transseptal cannulation.^8^ Transseptal needles and sheaths have also been paired with localization tools such as MediGuide™ (Abbott Laboratories, Chicago, IL, USA).

### Preprocedural mapping or planning

Preprocedural cross-sectional imaging, including predominantly computed tomography (CT), has been used to map atrial anatomy prior to ablation. Imaging of left atrial volumes and geometry allows for prognostication, improved electroanatomical map creation, and ruling out of the presence of pulmonary vein stenosis or left atrial appendage thrombus (CT with delayed imaging).^25^ However, CT imaging adds to the ionizing radiation burden for the patient undergoing complex arrhythmia ablation.

Improvements in magnetic resonance imaging (MRI) and further experience with cardiac MRI have increased its use in the assessment of atrial structure and ventricular structure and function. Four-dimensional (4D) MRI flow and delayed enhancement gadolinium may provide prognostic or structural information about the atrium prior to ablation. 4D flow also has been used to evaluate the movement of blood in the left atrium and left atrial appendage and may eventually predict the risk of developing left atrial appendage thrombus.^26^

MRI has not been shown to be superior to transesophageal echocardiography for the evaluation of left atrial appendage thrombus.^27^ MRI also lacks the spatial resolution of CT. Improving upon these limitations may increase the utilization of periprocedural MRI and reduce the ionizing radiation burden of CT use.

## Catheter manipulation

Outside of setup and access, diagnostic and ablation catheter manipulation represents the bulk of the diagnostic and therapeutic procedures. Knowledge of the catheter position within the heart and the tracking of it over time is essential for mapping arrhythmias, in pacing procedures, and when delivering lesion sets. Fluoroscopy is frequently used to verify the location of the catheter tip and shaft intraprocedurally. Several technologies have been developed for this purpose.

Electroanatomical mapping (EAM) systems (CARTO^®^; Biosense Webster, Diamond Bar, CA, USA and EnSite™ NavX™; Abbott Laboratories, Chicago, IL, USA) are the backbone of fluoroless complex arrhythmia cases. They have enabled real-time tracking of catheters within the heart, the creation of activation maps, and the recording of ablation lesion locations and other points of interest. Some 3D mapping systems have incorporated fluoroscopy images (UniVu; Biosense Webster, Diamond Bar, CA, USA) to maximize the utility of images and reduce redundant image acquisition.

Although a 3D system may reduce radiation exposure, 65% of centers have never used and only 8% always use EAM for typical atrioventricular nodal reentrant tachycardia interventions, respectively. EAM systems are rarely used for standard procedures, with a negative trend present during the previous three years.^12^

The combination of EAM systems and intravascular catheter and transseptal guidance with ICE has enabled the development of zero-fluoroscopy complex arrhythmia ablation techniques. These techniques have been tested for feasibility and safety with successful completion of all steps of the procedure without the wearing of lead gowns.^8^ However, this is true of zero-fluoroscopy procedures utilizing ICE and EAM only; with zero-fluoroscopy, the time for constructing the right atrial geometry is 5.5 minutes ± 2.6 minutes, the time for left atrium geometry is 22 minutes ± 10 minutes, and the time for CT registration is 19 minutes ± eight minutes.^28^

Nonfluoroscopic catheter visualization has also been achieved by overlying indicator markers on prerecorded cine images (MediGuide™; Abbott Laboratories, Chicago, IL, USA).^29^ This system triangulates a coil build into catheters and sheaths using a 3D electromagnetic field and registers it to the patient’s prerecorded cine loops. A magnetic reference on the patient’s chest allows for respiratory and cardiac motion correction.

MRI has been used for real-time visualization and catheter tracking for atrial flutter ablation with zero to highly reduced fluoroscopy usage.^30^ Beyond the mapping of provider-manipulated catheters, work is currently underway to develop ablation or diagnostic catheters driven by MRI to perform designated ablation tasks.^31^

The utilization of low fluoroscopy for most atrial arrhythmia ablations is increasingly becoming the standard in many EP laboratories. Direct visualization of mapping and ablation catheters with EAM is the primary reason for this, and many operators have become increasingly comfortable with this strategy, particularly if paired with contact force-sensing catheters. Cryoballoon atrial fibrillation does not allow for direct visualization of the balloon catheter. A combination of techniques, including pressure waveform monitoring for balloon occlusion of the pulmonary veins as well as ICE guidance with Doppler to assess for balloon occlusion leaks, have made this a fluoroscopy-free ablation strategy as well.

## Conclusion

In the modern EP laboratory, fluoroscopy continues to be a staple for several basic procedures and maneuvers. Advances in technologies used in cardiac rhythm management have allowed operators to progressively spare patients and themselves from harmful ionizing radiation.

Most centers have been slow to adopt many of the described low fluoroscopy techniques. The attribution of risks or adverse events due to ionizing radiation is difficult because of the long lead time inherent in the pathophysiology of cataracts, musculoskeletal injuries, and malignancies.

Monitoring of radiation is often collected in aggregate, and single providers can have exposures beyond recommended levels. In many centers, there is no reimbursement for low-fluoroscopy techniques. The next generation of electrophysiologists, having been trained in EAM, may be more inclined to adopt this for routine procedures or to attempt zero-fluoroscopy endpoints.

Low-hanging fruit include adjusting fluoroscopy settings (specifically frames per second or pulse dose) and the removal of the scatter grid. Using ultrasound access for subclavian veins for devices and using EAM for routine and complex procedures are other examples. Infrastructure requirements and cost concerns decrease the availability of other 3D guide systems for biventricular pacemaker implantation or complex arrhythmia ablation.

The likely limitations to adopting low to zero fluoroscopy in ablation include increased costs of acquiring newer technologies and experienced operators being more comfortable with a traditional fluoroscopic-guided technique. There is an ongoing multicenter registry of low to zero fluoroscopy in atrial fibrillation ablation.^32^ The objective is to illustrate that a busy and productive EP practice can function with low complication rates and high levels of procedural success using the previously described strategies. By making these techniques a matter of course, we can train the next generation of EP practitioners to break the cycle of fluoroscopy dependence. With further advancement in eliminating fluoroscopy during device implantation, one could imagine a future EP lab being designed and developed without the need for fluoroscopic equipment and associated support systems.

## References

[1] Picano E, Santoro G, Vano E (2007). Sustainability in the cardiac cath lab. Int J Cardiovasc Imaging..

[2] Klein LW, Miller DL, Balter S (2010). Occupational health hazards in the interventional laboratory: time for a safer environment. J Radiol Nurs..

[3] Anselmino M, Sillano D, Casolati D, Ferraris F, Scaglione M, Gaita F (2013). A new electrophysiology era: zero fluoroscopy. J Cardiovasc Med (Hagerstown)..

[4] Rogers DP, England F, Lozhkin K, Lowe MD, Lambiase PD, Chow AW (2011). Improving safety in the electrophysiology laboratory using a simple radiation dose reduction strategy: a study of 1007 radiofrequency ablation procedures. Heart..

[5] Finkelstein MM (1998). Is brain cancer an occupational disease of cardiologists?. Can J Cardiol..

[6] Hardell L, Mild KH, Påhlson A, Hallquist A (2001). Ionizing radiation, cellular telephones and the risk for brain tumours. Eur J Cancer Prev..

[7] Ector J, Dragusin O, Adriaenssens B (2007). Obesity Is a major determinant of radiation dose in patients undergoing pulmonary vein isolation for atrial fibrillation. J Am Coll Cardiol..

[8] Ferguson JD, Helms A, Mangrum JM (2009). Catheter ablation of atrial fibrillation without fluoroscopy using intracardiac echocardiography and electroanatomic mapping. Circ Arrhythmia Electrophysiol..

[9] Theocharopoulos N, Damilakis J, Perisinakis K, Manios E, Vardas P, Gourtsoyiannis N (2006). Occupational exposure in the electrophysiology laboratory: quantifying and minimizing radiation burden. Br J Radiol..

[10] Attanasio P, Schreiber T, Pieske B (2017). Pushing the limits: establishing an ultra-low framerate and antiscatter grid-less radiation protocol for left atrial ablations. Europace..

[11] Drury P, Robinson A (1980). Fluoroscopy without the grid: a method of reducing the radiation dose. Br J Radiol..

[12] Estner HL, Grazia Bongiorni M, Chen J (2015). Use of fluoroscopy in clinical electrophysiology in Europe: Results of the European Heart Rhythm Association Survey. Europace..

[13] Goldstein JA, Balter S, Cowley M, Hodgson J, Klein LW (2004). Interventional Committee of the Society of Cardiovascular Interventions. Occupational hazards of interventional cardiologists: Prevalence of orthopedic health problems in contemporary practice. Catheter Cardiovasc Interv..

[14] Marichal DA, Anwar T, Kirsch D (2011). Comparison of a suspended radiation protection system versus standard lead apron for radiation exposure of a simulated interventionalist. J Vasc Interv Radiol..

[15] Haussen DC, Van Der Bom IM, Nogueira RG (2016). A prospective case control comparison of the ZeroGravity system versus a standard lead apron as radiation protection strategy in neuroendovascular procedures. J Neurointerv Surg..

[16] Germano JJ, Day G, Gregorious D, Natarajan V, Cohen T (2015). A novel radiation protection drape reduces radiation exposure during fluoroscopy guided electrophysiology procedures. J Invasive Cardiol..

[17] Simons GR, Orrison WW (2004). Use of a sterile, disposable, radiation-absorbing shield reduces occupational exposure to scatter radiation during pectoral device implantation. Pacing Clin Electrophysiol..

[18] Danon A, Shurrab M, Nair KM (2015). Atrial fibrillation ablation using remote magnetic navigation and the risk of atrial-esophageal fistula: international multicenter experience. J Interv Card Electrophysiol..

[19] Jin QI, Pehrson S, Jacobsen PK, Chen XU (2016). Efficacy and safety of atrial fibrillation ablation using remote magnetic navigation: experience from 1,006 procedures. J Cardiovasc Electrophysiol..

[20] Errahmouni A, Latcu DG, Bun SS, Rijo N, Dugourd C, Saoudi N (2015). Remotely controlled steerable sheath improves result and procedural parameters of atrial fibrillation ablation with magnetic navigation. Europace..

[21] Maurer T, Sohns C, Deiss S (2017). Significant reduction in procedure duration in remote magnetic-guided catheter ablation of atrial fibrillation using the third-generation magnetic navigation system. J Interv Card Electrophysiol..

[22] Crowhurst J, Haqqani H, Wright D (2017). Ultra-low radiation dose during electrophysiology procedures using optimised new generation fluoroscopy technology. Pacing Clin Electrophysiol..

[23] Jones DG, Stiles MK, Stewart JT, Armstrong GP (2006). Ultrasound-guided venous access for permanent pacemaker leads. Pacing Clin Electrophysiol..

[24] Thibault B, Mondésert B, Macle L (2016). Reducing radiation exposure during CRT implant procedures: single-center experience with low-dose fluoroscopy settings and a sensor-based navigation system (MediGuide). J Cardiovasc Electrophysiol..

[25] Romero J, Husain SA, Kelesidis I, Sanz J, Medina HM, Garcia MJ (2013). Detection of left atrial appendage thrombus by cardiac computed tomography in patients with atrial fibrillation: a meta-analysis. Circ Cardiovasc Imaging..

[26] Markl M, Lee DC, Furiasse N (2016). Left atrial and left atrial appendage 4D blood flow dynamics in atrial fibrillation. Circ Cardiovasc Imaging..

[27] Mohrs OK, Nowak B, Petersen SE (2006). Thrombus detection in the left atrial appendage using contract-enhanced MRI: a pilot study. Am J Roentgenol..

[28] Reddy VY, Morales G, Ahmed H (2010). Catheter ablation of atrial fibrillation without the use of fluoroscopy. Heart Rhythm..

[29] Sommer P, Rolf S, Piorkowski C (2014). Nonfluoroscopic catheter visualization in atrial fibrillation ablation experience from 375 consecutive procedures. Circ Arrhythm Electrophysiol..

[30] Hilbert S, Sommer P, Gutberlet M (2016). Real-time magnetic resonance-guided ablation of typical right atrial flutter using a combination of active catheter tracking and passive catheter visualization in man: initial results from a consecutive patient series. Europace..

[31] Greigarn T, Cavuşoğlu MC (2014). Task-space motion planning of MRI-actuated catheters for catheter ablation of atrial fibrillation. Rep U S..

[32] Zei P, Thosani A, Mitra R (2017). Minimal fluoroscopy atrial fibrillation catheter ablation: a prospective multicenter registry (P1405). Europace..

